# Extended hydrogen breath test analysis for optimized diagnosis of SIBO-positive IBS patients

**DOI:** 10.1186/s12876-026-04782-w

**Published:** 2026-04-06

**Authors:** A Dahlgren, P Grybäck, H Jacobsson, PM Hellström

**Affiliations:** 1 Department of Medical Sciences, Uppsala University, Uppsala, Sweden; 2 Department of Hospital Physics and Nuclear Medicine, Karolinska University Hospital, Solna, Sweden

**Keywords:** Hydrogen, IBS, Lactulose, Microbiota, Small intestinal bacterial overgrowth, SIBO

## Abstract

**Background:**

Small intestinal bacterial overgrowth (SIBO) is suggested in irritable bowel syndrome (IBS). Optimal hydrogen levels and time frame for diagnosing SIBO are still under discussion. Our primary aim was to consolidate a discriminating threshold for a positive lactulose hydrogen breath test (LHBT) in IBS. As a secondary aim, we optimized the diagnostic time frame for the small bowel.

**Methods:**

LHBT was performed on 503 subjects who met the inclusion criteria. After excluding non-hydrogen producers, the remaining 462 subjects were 92 healthy individuals and 370 IBS patients. Peak hydrogen levels were summarized as median values with interquartile range.

**Results:**

At the 80-min orocecal cut-off, healthy subjects showed a peak hydrogen of 8(4–11) ppm compared with 10(4–29) ppm in the overall IBS group (p <0.0001). Using ≥20 ppm cut-off, sensitivity was 38% and specificity 77%. Peak hydrogen was highest in IBS-D (24(9-40) ppm; p <0.0001), intermediate in IBS-M (7(4-14) ppm), and lowest in IBS-C (7(4-10) ppm), showing sensitivities of 61%, 23%, and 10%, respectively, with specificity 77%.After antibiotics, IBS patients with low hydrogen were unchanged, whereas most with high hydrogen reduced their hydrogen levels (p <0.01).

**Conclusion:**

Using a cut-off level of ≥20 ppm during the first 80 minutes, LHBT can diagnose SIBO in people with IBS showing high breath hydrogen, as compared with those having low breath hydrogen. Hence, SIBO-positive patients can be separated from SIBO-negative IBS patients. To this end, a majority of SIBO-positive subjects respond to antibiotic treatment.

## Key Summary

• The lactulose hydrogen breath test can be used to diagnose SIBO-positive IBS patients.

• A diagnostic cut-off level of 20 ppm hydrogen within 80 minutes indicates better diagnostic criteria for bacterial overgrowth.

• A substantial proportion of IBS-D patients have high hydrogen emanating from the small intestine indicating small intestinal bacterial overgrowth.

## Introduction

Accumulating evidence suggests a potential role for dysbiosis in irritable bowel syndrome (IBS) [1–3]. Small intestinal bacterial overgrowth (SIBO) is detected in up to 78% of IBS cases [4]. This sizable prevalence may be attributed to differences in ethnicity, microbiology, and various diagnostic criteria employed. Non-invasive breath tests have gained interest due to the ease and timeliness with which an SIBO diagnosis can be made. Still, bacterial cultures of aspirate from the proximal jejunum are described as the gold standard for diagnosing SIBO [5]. However, this has been challenged because it carries a risk of sampling error if not multiple regions of the small bowel are sampled, and the cut-off level for SIBO has been discussed [6, 7]. In addition, there is a major contamination risk at sampling, and commonly the sampled bacteria are not culturable; therefore, this diagnostic method is suboptimal [5].

Different types of breath tests have been employed but are hampered by the lack of a true standard for the performance and interpretation of data. Depending on the chosen fermentable bacterial substrate and the timing of readouts and diagnostic hydrogen cut-off levels, various studies arrive at different recommendations for diagnosing SIBO. This has led to interactive working groups advocating essential rules for employing hydrogen breath tests for diagnostic purposes [8–10].

The lactulose hydrogen breath test (LHBT) is clinically used as a non-invasive proxy for the diagnosis of SIBO. A key limitation of LHBT interpretation is the timing of hydrogen measurement, as under physiological conditions, only minimal hydrogen is detectable in breath until fermentation occurs in the cecum, which harbors a high bacterial load. Studies in healthy individuals demonstrate an oro-cecal transit time typically ranging between 72 and 90 min [11–13].

Accordingly, the North American Consensus guidelines recommend a diagnostic cut-off time of 90 min for LHBT interpretation [8], whereas European guidelines propose shorter cut-off times, in some cases as early as 40 min, to reduce false-positive results related to rapid colonic transit, albeit at the expense of sensitivity [9].

With respect to diagnostic hydrogen thresholds, several cut-off levels have been proposed for SIBO diagnosis [8, 9]. A rise, equal to or greater than (≥) 20 ppm in breath hydrogen, has been adopted in expert consensus recommendations as a standardized criterion, largely to favor the specificity of the test. However, comparative studies demonstrate only modest concordance between breath testing and small bowel aspirate culture [14], underscoring the absence of a robustly validated hydrogen cut-off.

The present study was therefore conducted to optimize diagnostic hydrogen cut-off values for SIBO in patients with irritable bowel syndrome (IBS). Using an optimized transit time framework, breath hydrogen levels were compared between healthy controls and patients with IBS, which were further stratified across different subgroups of IBS.

## Materials and methods

### Ethical considerations

The need for consent to participate was deemed unnecessary by the Institutional Review Board (IRB) according to national regulations for anonymized data. The study was conducted in accordance with the principles of the Declaration of Helsinki. The study was approved by the Uppsala Institutional Review Board (2022-04646-01); all patients’ identities anonymized.

### Lactulose hydrogen breath test

In a retrospective observational analysis, a total of 503 eligible subjects that underwent a LHBT were evaluated. Eligibility required absence of lactase or sucrase deficiency, celiac disease, bile acid malabsorption, inflammatory bowel disease (including ulcerative colitis, Crohn’s disease, collagenous or lymphocytic colitis), gastrointestinal malignancy, or clinical signs of maldigestion. Forty-one individuals were identified as hydrogen non-producers (0 ppm hydrogen in breath) and excluded from further analysis. The remaining 462 subjects were classified according to the Rome III criteria into 92 healthy controls (44 men, 48 women, 19–81 years of age), and 370 patients diagnosed with IBS (156 men, 214 women, 19–77 years of age; 166 diarrhea-dominant (IBS-D), 135 mixed type (IBS-M) and 69 constipation-dominant (IBS-C) [15].

The LHBT was carried out using breath sampling equipment with an electrochemical hydrogen-sensitive cell (GMI Medical Ltd, Renfrew, UK) with a resolution of 1 ppm, accuracy ± 2 ppm, and linear range 2-150 ppm.

After an overnight fast from 8:00 PM, breath sampling was performed the following morning at 8:00–10:00 AM. Two baseline end-expiratory breath samples were collected at -10 and 0 min. Thereafter, 10 g of lactulose solution (670 mg/mL; Laktulos Meda, Stockholm, Sweden) was ingested, and breath samples were collected every 10 min over the following 180 min. During the breath sampling period, the participants were not allowed to exercise, drink, eat, or smoke, as interference may cause falsely high hydrogen levels. Drug treatment with antibiotics, dopamine receptor blockers or opioid receptor agonists, proton pump inhibitors, and laxatives was discontinued at least 28 days before the LHBT was carried out.

The breath hydrogen concentration of each subject was plotted as the hydrogen concentration (ppm) against time (min) over the assessment period and measured against baseline. Peak hydrogen values were used to calculate diagnostic accuracy [16]. In a subset of 109 IBS patients, data on antibiotic administration and re-testing after 14 days of treatment were obtained. Symptoms characterizing subgroups of IBS were obtained from clinical notes.

### Statistics

Values are presented as medians with interquartile range and the 5–95% percentiles or means and 5–95% confidence interval within parentheses where appropriate.

Differences between the study groups were calculated using the healthy control group as comparator. Because breath hydrogen values showed a non‑normal distribution (Anderson–Darling normality test, *p* < 0.001), continuous variables are presented as median and interquartile range (IQR). Differences between groups were analyzed using the non‑parametric Kruskal–Wallis test with Dunn’s post‑hoc comparisons.

Receiver operating characteristics (ROC) were calculated to set the cut-off points and accuracy of the LHBT, including sensitivity and specificity. Statistical overlap between the different study groups was calculated by linear-scale kernel density estimates employing the Kolmogorov-Smirnov test.

## Results

After challenge with lactulose, the healthy control group had a mean oro-cecal transit time (OCTT) of 97 (92–102) minutes until the 20 ppm hydrogen level in breath occurred. OCTT of the whole IBS group was 92 (88–96) minutes (ns). The lower limit of the 95% confidence interval of the LHBT transit time in healthy controls showed transit times of 0–80 min as evaluable boundaries for the assessment of hydrogen production in the small intestine (cf.16).

**Fig. 1:**
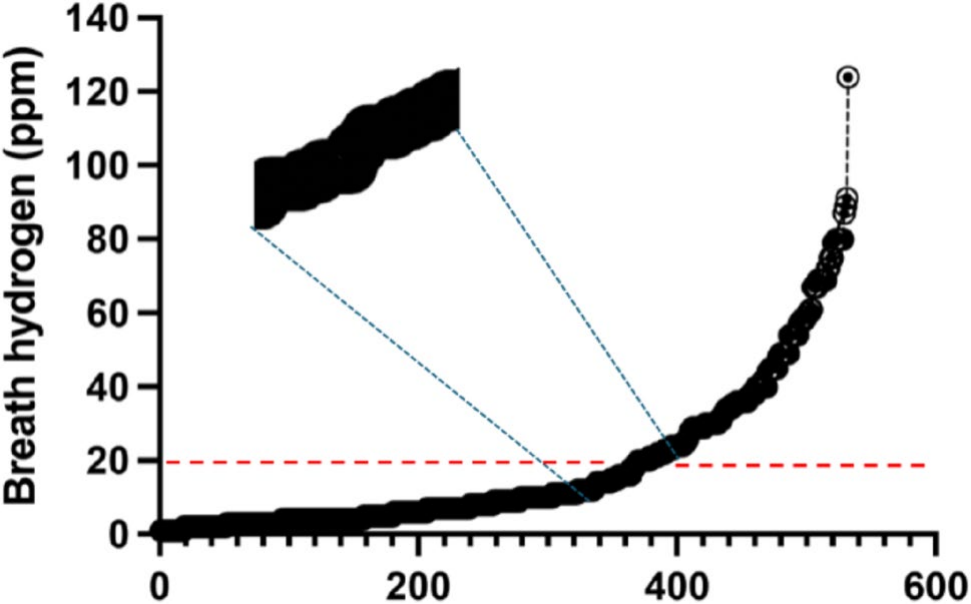
Breath hydrogen lineup of all subjects included in the study (*n* = 462). Red dashed line indicates 20 ppm cut-off for breath hydrogen. Insert shows detail of the indent in the curve at the diagnostic level 20 ppm

The LHBT showed expected hydrogen values from 1 ppm to 124 ppm in the whole study group within the 80-minute assessment time frame corresponding to the small bowel (Fig. 1).

**Fig. 2:**
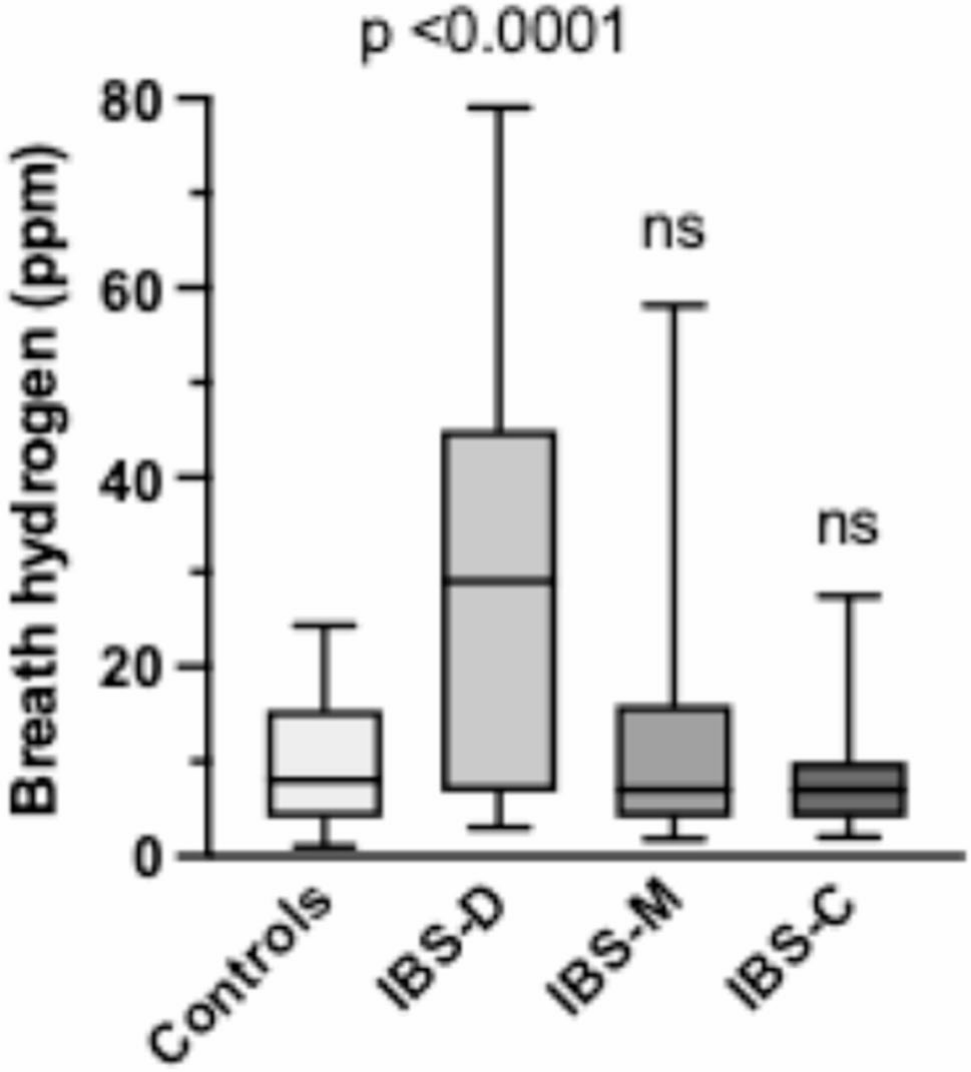
Breath hydrogen levels in healthy controls (*n* = 92) compared with IBS-D (*n* = 166), IBS-M (*n* = 135), and IBS-C (*n* = 69). Data shown as medians and IQR with 5–95 percentiles; ns, non-significant

Apparent differences between the IBS subgroups were found by applying the ≥20-ppm hydrogen level and 80-minute readout time as diagnostic cut-offs. Median peak breath hydrogen concentrations differed significantly between the IBS subtypes (*p* < 0.0001), where IBS-D with high hydrogen levels was clearly separated from controls (*p* < 0.0001), while IBS-M and IBS-C were not (Fig. 2).

The accuracy of LHBT for the entire IBS group versus controls was evaluated using the 60- and 80-minute OCTT readouts and the ≥20 ppm cut-off. Results showed a better diagnostic accuracy of the 80-minute readout with a sensitivity of 38% at 80 min compared with 26% at 60 min; both with specificity 77%.

The diagnostic performance of the LHBT was further evaluated for the different IBS subgroups IBS-D, IBS-M and IBS-C, employing the 80-minute readout time and ≥ 20 ppm cut-off level. The diagnostic accuracy and odds ratio for IBS-D were superior to those of IBS-M and IBS-C. Additional diagnostic features, such as sensitivity and specificity, as well as positive and negative predictive values, were also better for IBS-D than for IBS-M and IBS-C (Tables 1 and 2).

**Table 1:** Diagnostic performance of the lactulose hydrogen breath test over 60 and 80 min with a hydrogen cut-off ≥ 20 ppm in patients with irritable bowel syndrome (IBS; *n* = 370) versus healthy controls (Controls; *n* = 92)

Comparison (time-matched)	Sensitivity (%)	Specificity (%)	Odds Ratio (95% CI)
60 min: All IBS vs. Controls	27	77	1.24 (0.72–2.12)
80 min: All IBS vs. Controls	38	77	2.03 (1.20–3.46)

*CI* Confidence interval

**Table 2:** Diagnostic performance of the lactulose hydrogen breath test over 80 min with a hydrogen cut-off ≥ 20 ppm for different IBS subgroups. Healthy controls (*n* = 92) as reference

IBS subgroup	Sensitivity (%)	Specificity (%)	PPV (%)	NPV (%)	Accuracy (%)	Odds Ratio (95% CI)
IBS-D (*n* = 166)	61	77	83	52	67	5.25 (2.95–9.36)
IBS-M (*n* = 135)	23	77	60	41	45	1.01 (0.54–1.89)
IBS-C (*n* = 69)	10	77	25	53	48	0.38 (0.15–0.96)

*IBS* Irritable bowel syndrome, *IBS-D* Diarrhea-dominated IBS, *IBS-M* Mixed IBS, *IBS-C* Constipation-dominated IBS. Specificity identical across analyses due to the fixed control false-positive rate. *CI* Confidence interval

### Small intestinal bacterial overgrowth in different patient groups

The median peak and IQR breath hydrogen level was 8 [4–11] ppm in healthy controls using the 80-minute readout interval. In the whole IBS group, the median and IQR breath hydrogen level was 10 [4–28] ppm (*p* < 0.0001 vs. controls). The median peak values and IQR of the different subgroups were as follows: IBS-D 24 (9–40) ppm (*p* < 0.0001), IBS-C 7 [4–10] ppm (ns) and IBS-M 7 [4–14] ppm (ns), all versus control.

**Fig. 3:**
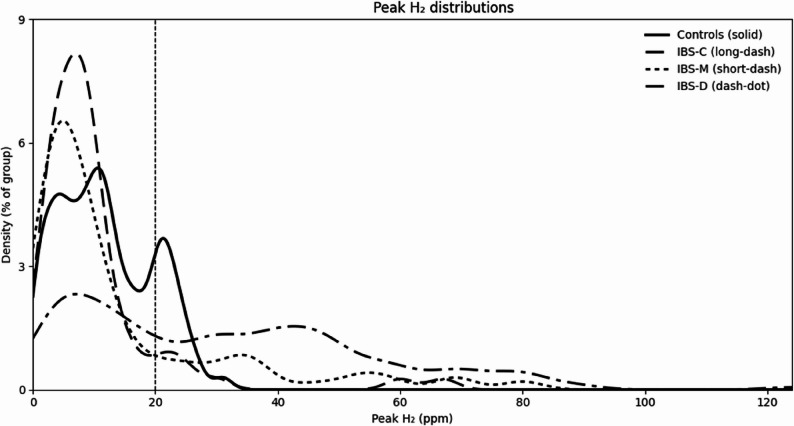
Linear-scale kernel density estimates of peak breath hydrogen concentrations in healthy controls and the IBS subtypes. Distributions are normalized to the percentage of subjects per group. The abscissa shows 0 to 124 ppm with 20-ppm intervals. The ordinata is scaled in 3% increments of the tested population

Using linear-scale kernel density estimates, the hydrogen overlap between healthy controls and IBS-D was 46%, whereas the overlap with IBS-M was 71% and IBS-C was 82% (Fig. 3), reflecting a marked shift of peak hydrogen values towards higher values in IBS-D. Hence, high breath hydrogen was predominantly associated with IBS-D as compared with IBS-M and IBS-C. Among 139 patients classified with high breath hydrogen ≥ 20 ppm, 102 (73%) were recovered in the IBS-D subgroup, while 31 (22%) were found in the IBS-M subgroup and 6 (4%) in the IBS-C subgroup, indicating a strong concordance between high hydrogen production and IBS-D.

**Fig. 4:**
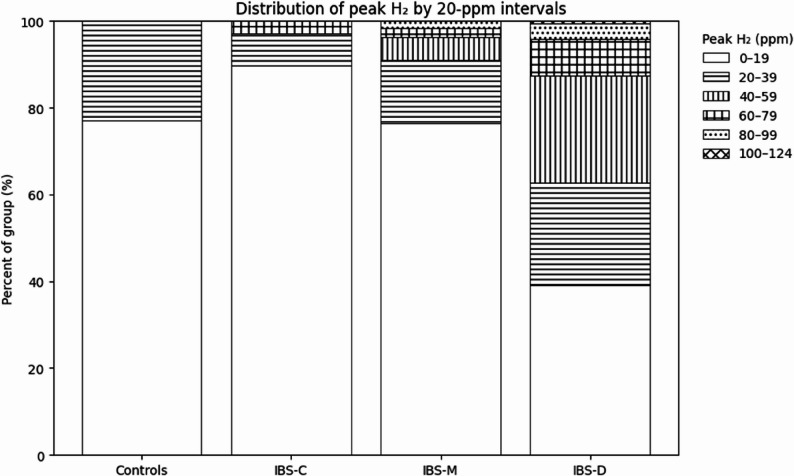
Distribution of peak breath hydrogen concentrations by 20-ppm intervals, expressed as percentage of subjects per IBS subgroup. Percentages derived from the area under linear-scale kernel density estimates

The breath hydrogen results across all diagnostic subgroups showed that IBS-D retained substantial bacterial hydrogen production compared with IBS-M and IBS-C, (Fig. 4).

### Response to antibiotics

As an ad hoc observation, a subset of 109 patients was given antibiotics and re-tested after 14 days of treatment. In the high hydrogen group 31 out of 39 showed a significant reduction of their breath hydrogen levels (*p* < 0.01). Abdominal symptoms improved in 22. Among 70 patients with low breath hydrogen, none showed any clear change of their breath hydrogen levels, whereas few had mitigated symptoms.

## Discussion

The hydrogen breath test is an accepted non-invasive clinical method to diagnose SIBO because only the microbiota, but not mammalian cells, can produce hydrogen [19]. We found that among patients with IBS, primarily patients with IBS-D are capable of producing high breath hydrogen during the passage of lactulose through the small bowel. For the IBS-M and IBS-C, correspondingly fewer people produce high amounts of hydrogen. Because breath hydrogen concentrations demonstrated a right‑skewed distribution, results were verified using non‑parametric statistical testing. The observed group differences remained statistically significant, confirming the robustness of the findings. Hence, our data speaks in favour of SIBO as a possible diarrheogenic component in IBS-D.

We found that our results are in line with those of the North American consensus for breath testing, which recommends ≥20 ppm hydrogen level and 90-minute readout time for diagnosis [8], while the European guidelines do not specify the diagnostic readout time and hydrogen breath levels required for diagnosis [9]. Based on our previous study [16], we chose a ≥20 ppm increase of hydrogen concentration from baseline within 80 min for diagnosis. The 80-minute readout is a close approximation to the North American consensus on breath hydrogen interpretation, and at the same time minimizes the risk of over-diagnosing SIBO merely because of rapid transit. Across investigations employing the LHBT, the IBS-D subtype consistently exhibits the highest prevalence of hydrogen-positive SIBO in line with our results [17, 18]. A study by Rana and Malik [20] showed that IBS-D patients were predominantly hydrogen-positive, compared with those with IBS-C, while IBS-M showed intermediate results. Later systematic reviews confirmed this tendency, indicating that hydrogen-dominant SIBO occurs preferentially in the diarrhea-predominant IBS phenotype [21]. Although the LHBT may produce false positive results in individuals with rapid oro-cecal transit, this methodological concern does not fully account for the robust hydrogen responses typically observed in IBS-D. Collectively, evidence supports the view that accelerated intestinal transit and small bowel bacterial overgrowth may coexist in a subset of IBS-D patients, suggesting that hydrogen-dominant SIBO represents a key pathophysiological characteristic of diarrhea-predominant IBS [21].

Calculations of the diagnostic accuracy showed that the sensitivity and specificity was optimal using the ≥20-ppm cut-off, with a limited risk of over-diagnosing SIBO. Hence, this cut-off level was used for further diagnostics, in line with the American consensus [8]. Applying a 60-minute cut-off level, the calculated odds ratio was close to unity, indicating limited discriminatory value at this early time point under conditions of a high false positive rate in controls. In contrast, the 80-minute odds ratio was approximately two-fold, supporting a statistically meaningful association between a positive breath hydrogen test and the IBS status at this time point. Our findings reinforce the fact that hydrogen elevations observed at 80 min provide greater diagnostic relevance than those obtained at 60 min.

Employing the 80-minute readout time frame and ≥20 ppm as the cut-off for a proper diagnosis of SIBO, we found that 37% of patients clinically diagnosed with IBS were found to have SIBO. This upholds earlier findings of SIBO in the presence of IBS, in the range of 30–85% of patients with IBS symptoms [20, 22–25]. However, in our hands the number of patients diagnosed with IBS-D represented 73% of the IBS with high hydrogen. This finding suggests a pathophysiological importance of SIBO in IBS-D-like symptoms commensurate with our present findings. However, because SIBO is a complex condition that commonly includes not only symptoms from the gastrointestinal tract, but also malnutrition, neuropathy, muscular atrophy and cachexia, a personalized view on the treatment is mandatory [7, 26].

## Strengths and weaknesses

Data were randomly extracted from LHBT records performed for clinical suspicion of SIBO and are therefore considered representative of real-world clinical practice. Symptom reporting may nonetheless be affected by recall bias, despite efforts to clarify uncertain clinical information.

Healthy volunteers were included as a comparator to provide normative reference data. Although age may confound SIBO prevalence, this limitation has been well described in both pathophysiological and methodological studies [27].

Lactulose was used as a biomarker in this study because it remains unchanged throughout the small intestine, thereby enhancing the sensitivity of detection for bacterial overgrowth in both the proximal and distal regions of the gut. However, a rapid transit time could be misdiagnosed as SIBO if it reaches the colon sooner than the 80-minute cut-off time. Thus, our finings support a cut-off time of 80 min to reduce false positives. In line with this, the European guidelines recommend a standard cut-off timing of 60 min to avoid false positives [9], however, at the cost of false negatives.

Elevated breath hydrogen is a marker of intestinal carbohydrate fermentation, which has been associated with diarrhea and accelerated gut transit [28, 29]. Experimental data suggest that hydrogen itself can modulate motility, particularly in the proximal colon where fermentation and hydrogen production predominates [30]. Clinically, symptom improvement after rifaximin in non-constipated IBS and breath test positive patients occurs even when breath hydrogen does not fully normalize, underscoring that hydrogen is not a single causative factor, but an imperfect marker of fermentation-driven mechanisms [31, 32].

One limitation of our study is that only hydrogen was measured, and excluding hydrogen non-producers without methane measurements may bias the estimation of SIBO prevalence in IBS. Also, the absence of an independent gold standard limits our ability to validate the diagnostic cut-off. The value of a jejunal aspirate has been questioned since it is technically difficult to perform and beyond daily clinical practice [33].

In conclusion, LHBT is capable of diagnosing SIBO-positive IBS, provided a validated readout time frame and a verified peak hydrogen cut-off level are applied. It is suggested that IBS patients can be subdivided into those who are low or high in breath hydrogen, where high breath hydrogen commonly reflects SIBO manifesting as chronic diarrhea.

## Data Availability

The data underlying this presentation is available as pseudonymized human data through the guarantor of the article Prof Per M. Hellström, Department of Medical Sciences, Uppsala University, Uppsala, Sweden.
